# Influence of lifestyle on stroke risk among adults over 40 years in northern China: A retrospective case-control study

**DOI:** 10.1097/MD.0000000000045707

**Published:** 2025-11-07

**Authors:** Jiantao Yu, Jie Li, Huiyong Yu, Qiang Zhou

**Affiliations:** a Hebei Medical University Second Hospital, Shijiazhuang, Hebei, China; b Hebei Medical University First Hospital, Shijiazhuang, Hebei, China; c Hebei Medical University Third Hospital, Shijiazhuang, Hebei, China.

**Keywords:** eating habits, lifestyle, risk factors, stroke

## Abstract

This study investigates the association between lifestyle factors and stroke occurrence based on health examination data of community residents aged ≥ 40 years. From July 2020 to November 2020, we collected data from 2 communities in Shijiazhuang using convenience sampling combined with cluster sampling. Demographic information, physical examination results, and laboratory test data were collected via the Community and Township Population Cardiovascular and Cerebrovascular Disease Risk Factor Screening Form. We used multiple binary logistic regression analysis to assess the associations between different lifestyle factors and stroke risk, adjusting for potential confounding variables. The prevalence of stroke among community residents aged ≥ 40 years in Shijiazhuang was 2.27%. Significant differences were observed between stroke patients and non-stroke participants in terms of eating habits, fruit and vegetable intake, physical inactivity, overweight status, and smoking behavior. After adjusting for confounding factors, adequate fruit and vegetable intake and a balanced meat-vegetable diet were associated with a protective effect against stroke. Smoking, overweight, and physical inactivity were identified as independent risk factors for stroke. No significant association was found between alcohol consumption or salt taste preference and stroke risk. Compared with participants with 0 to 1 unhealthy lifestyle factors, the stroke risk was 1.75-fold, 2.70-fold, and 22.67-fold higher in those with 2 to 3, 4 to 5, and ≥6 unhealthy lifestyle factors, respectively. Healthy lifestyles play a crucial role in stroke prevention. Future studies should further investigate optimal dietary patterns and lifestyle habits for reducing stroke risk, considering individual nutritional needs and preferences.

## 1. Introduction

Stroke is an acute cerebrovascular disease characterized by high morbidity, disability, and mortality, whose main clinical manifestations are ischemic or hemorrhagic brain tissue injury. With global population aging and lifestyle transitions, stroke has become one of the leading causes of death and long-term disability worldwide, imposing heavy epidemiological and socioeconomic burdens on healthcare systems.^[1]^ The World Health Organization (WHO) estimates that by 2030, the incidence of stroke in low- and middle-income countries could account for 80% of global stroke incidence.^[2]^ Globally, stroke is currently the second leading cause of death after ischemic heart disease in most European and American countries.^[3]^ In China, the incidence of stroke has been increasing continuously at an annual rate of 5.4%.^[4]^ Therefore, as a major public health challenge, stroke prevention and treatment have become a national policy priority in China.

According to the American Heart Association and American Stroke Association’s guidelines, controlling the risk factors of stroke is an effective strategy for preventing stroke.^[1]^ While non-modifiable factors (e.g., age, gender, genetic predisposition) contribute to stroke susceptibility, lifestyle factors – including dietary patterns, smoking, alcohol consumption, physical activity, and body weight management – have gained increasing attention for their potential role in stroke prevention, given their modifiable nature.^[2–4]^ The results of the “Interstroke” study from 32 countries showed that 96.7% of stroke cases were related to interventional risk factors worldwide.^[5]^ Although numerous epidemiological studies have explored associations between individual lifestyle behaviors and stroke risk,^[6–10]^ conflicting conclusions persist across different populations and study designs.

For example, some cohort studies have reported that adherence to a healthy diet (e.g., high fruit and vegetable intake) is associated with reduced stroke risk,^[11]^ while others have failed to detect such an association. This discrepancy may be attributed to differences in dietary assessment methods or confounding factors such as participant age.^[12,13]^ A prospective epidemiological study involving over 135,000 participants from 18 countries found that higher intake of fruits, vegetables, and legumes was associated with lower total mortality, but yielded inconsistent effects on stroke risk after multivariable adjustment.^[14]^ Second, the magnitude of impact of specific lifestyle factors – including alcohol consumption, body mass index (BMI), and physical activity – remains some debated among researchers. Regarding drinking habits, Zhang et al^[15]^ identified a “J-shaped curve” via a dose-response meta-analysis of prospective studies, suggesting that light alcohol intake may confer a protective effect against ischemic stroke. However, another study demonstrated a linear dose-response relationship between alcohol intake and hemorrhagic stroke.^[16]^ For physical activity, a population-based cohort study in Ningbo, China, found that among adults aged ≥ 40 years, high-intensity physical activity was associated with a 63.1% lower stroke risk compared with low-intensity activity, whereas moderate activity showed no significant protective effect.^[17]^ In contrast, international guidelines propose that even moderate activity reduces stroke risk by improving vascular endothelial function.^[18]^

Regarding weight management, the traditional view holds that overweight and obesity are independent risk factors for stroke.^[19]^ However, a meta-analysis testing the “obesity paradox” hypothesis found that mild overweight was associated with lower post-stroke mortality.^[20]^ Additionally, waist circumference has been suggested to be a stronger predictor of stroke risk than BMI in Chinese populations.^[21]^

Most existing studies investigating the association between lifestyle and stroke have focused on hospital-based populations, with limited attention paid to community-dwelling adults and a marked lack of analysis on the combined effects of multiple lifestyle factors. Our study aims to address these gaps by comprehensively analyzing lifestyle behaviors among community residents aged ≥ 40 years. Through this work, our findings may help refine current stroke prevention guidelines and provide a more nuanced understanding of lifestyle choices that can mitigate stroke risk.

## 2. Study subjects

From July 2020 to November 2020, we employed a combination of convenience sampling and cluster sampling to select participants from 2 communities in Hebei Province. Inclusion Criteria: age ≥ 40 years old; permanent residents of the community (living for 6 months or more); and Inform participants of the purpose of the study, and voluntarily participate and sign an informed consent form. Exclusion Criteria: severe systemic diseases or malignant tumors; hearing impairment, inability to communicate normally, and inability to cooperate with the investigation; cognitive impairment or psychiatric disorders; and confined to bed for years, unable to fully act.

## 3. Date collection

Trained and qualified community medical staff collected study data from participants using the Cardiovascular and Cerebrovascular Disease Risk Factor Screening Form. This form included 2 main components: general information and physical examination data. General information was collected by community medical staff via face-to-face interviews, covering demographic details (sex, age, marital status, educational level, annual household income), family history of stroke, and lifestyle factors. Physical examination and laboratory test data were obtained through centralized health checkups. The physical examination included measurements of height, weight, heart rate, systolic blood pressure (SBP), and diastolic blood pressure (DBP). Laboratory tests included assessments of fasting blood glucose (FBG), triglycerides (TG), total cholesterol (TC), low-density lipoprotein cholesterol (LDL-C), and high-density lipoprotein cholesterol (HDL-C). Imaging examinations consisted of abdominal ultrasound, chest radiography, and electrocardiography.

## 4. Disease diagnostic criteria

Stroke status was determined using a combination of self-reported prior stroke diagnoses and neurological assessment by a specialist in accordance with WHO criteria.^[22]^ Self-reported stroke history was ascertained via the question: “Have you ever been told by a doctor or other healthcare professional that you had a stroke?” Participants who answered “yes” were asked to provide details including symptoms, onset date, medical records, and imaging data to verify the original diagnosis. For this study, stroke was defined as subarachnoid hemorrhage, intracerebral hemorrhage, or cerebral ischemic necrosis; transient ischemic attack, stroke secondary to brain tumor, brain metastasis, or trauma were excluded. Diagnostic criteria for comorbidities were as follows: Hypertension: SBP ≥ 140 mm Hg and/or DBP ≥ 90 mm Hg, or SBP < 140 mm Hg and DBP < 90 mm Hg with antihypertensive medication use or a self-reported history of hypertension.^[23]^ Diabetes mellitus: FBG ≥ 7.0 mmol/L, or FBG < 7.0 mmol/L with hypoglycemic medication use or a self-reported history of diabetes.^[24]^ Hyperlipidemia: TC ≥ 6.2 mmol/L, LDL-C ≥ 4.1 mmol/L, TG ≥ 2.3 mmol/L, or HDL-C < 1.0 mmol/L.^[25]^ Atrial fibrillation: Confirmed by self-reported history of atrial fibrillation or on-site electrocardiogram findings.

## 5. Lifestyle

Smoking status was categorized into 3 groups: never smoker, former smoker, and current smoker. Current smoking was defined as self-reported smoking of more than one cigarette per day for a continuous or cumulative period of 6 months. Overweight was defined as a BMI ≥ 24, where BMI was calculated as weight in kilograms divided by the square of height in meters (kg/m^2^); Alcohol consumption frequency was categorized into 3 groups: never drinker, light drinker, and heavy drinker. Heavy drinking was defined as consuming more than 100 g of white wine at least 3 times per week; Exercise intensity was defined in accordance with the standardized exercise intensity classification criteria established by the WHO and the American College of Sports Medicine.^[26]^ Sufficient physical activity was defined as engaging in moderate or vigorous exercise for ≥30 minutes per session, on ≥3 occasions per week. Specifically: Moderate exercise: Activity corresponding to 3.0 to 5.9 metabolic equivalents (METs), 64 to 76% of maximum heart rate, or activity that causes faster but regular breathing and the ability to speak in complete sentences (e.g., brisk walking, casual cycling); Vigorous exercise: Activity corresponding to ≥6.0 METs, 77 to 93% of maximum heart rate, or activity that causes deep, rapid breathing and the ability to speak only in short phrases (e.g., running, intense swimming); Salt taste preference was categorized into 3 groups: light, moderate, and salty; Dietary patterns based on vegetable and meat consumption were classified into 3 types: Vegan: No animal products consumed. Meaty: Meat-dominant diet with minimal vegetable intake. Balanced: Roughly equal proportions of meat and vegetables; Quantitative intake of fruits and vegetables was each categorized by weekly frequency of meeting a ≥100 g daily threshold: <2 days/week, 3 to 4 days/week, or >5 days/week.

## 6. Statistical analysis

Statistical analyses were performed using SPSS 26.0 software (IBM SPSS Statistics for Windows). Categorical variables were presented as frequencies (n) and percentages (%). The χ^2^ test or Fisher exact test was used to compare general characteristics and lifestyle factors between stroke patients and non-stroke participants, with Bonferroni correction applied for post hoc pairwise comparisons. Multiple binary logistic regression models were constructed using the forced entry method to calculate odds ratios and their corresponding 95% confidence intervals (CIs), aiming to explore the multivariable associations between lifestyle factors and stroke risk. All hypothesis tests were two-tailed, and statistical significance was set at *P* < .05.

## 7. Result

### 7.1. Basic information about the research objects

A total of 3734 subjects were included in this study, ranging from 40 to 95 years old, with an average age of 59.58 ± 9.36, including 1481 males (39.66%). 2253 females (60.33%). A total of 85 patients (2.27%) with ischemic stroke and hemorrhagic stroke. The education level of the subjects: Primary school and below was 654 cases (17.51%), Junior high school and technical secondary school level was 2585 (69.22%) cases, College degree and above was 495 (13.25%) cases. Family income: There were 988 cases (26.45%) with annual income <5000, 220 cases (5.89%) with annual income between 5000–10,000, 364 cases (9.74%) with annual income between –20,000, and a total of 2162 cases (57.90%) with annual income >20,000. There were 1407 patients with hypertension (37.68%), 558 patients with diabetes (14.94%), 1920 patients with dyslipidemia (51.41%), 79 patients with coronary heart disease (2.11%), and 30 patients with prior atrial fibrillation (0.80%) in the study population. A total of 393 patients (10.52%) with carotid artery stenosis were found by carotid ultrasound examination (Table 1).

**Table 1:** General demographic characteristics.

	Stroke N = 85		No-stroke N = 3649		χ^2^	*P*
	N	%	N	%		
Age						
40–64	11*	12.90%	1875*	51.40%	49.74	**<.001**
65–79	67†	78.80%	1640†	44.90%		
>80	7†	8.20%	134†	3.70%		
Sex						
Male	44*	51.80%	1437*	39.40%	5.32	**.021**
Female	41†	48.20%	2212†	60.60%		
Educational level						
Primary school and below	27*	31.80%	627*	17.20%	12.23	**.002**
Junior high school	49†	57.60%	2536†	69.50%		
College degree and above	9†	10.60%	486†	13.30%		
Income						
<5000	29	34.10%	959	26.30%	4.15	.246
5000–10,000	7	8.20%	213	5.80%		
10,000–20,000	8	9.40%	356	9.80%		
>20,000	41	48.20%	2121	58.10%		
Hypertension						
No	26*	30.60%	2301*	63.10%	37.29	**<.001**
Yes	59†	69.40%	1348†	36.90%		
Diabetes						
No	59*	69.40%	3117*	85.40%	16.75	**<.001**
Yes	26†	30.60%	532†	14.60%		
Hyperlipemia						
No	27	31.80%	1787	49.00%	9.84	**.002**
Yes	58	68.20%	1862	51.00%		
Family history of stroke						
No	53	62.40%	3272	89.70%	63.54	**<.001**
Yes	32	37.60%	377	10.30%		
Carotid artery stenosis						
No	67	78.80%	3274	89.70%	10.47	**<.001**
Yes	18	21.20%	375	10.30%		
Atrial fibrillation						
No	82	96.5%	3622	99.30%	8.11	**.004**
Yes	3	3.5%	27	0.70%		
Coronary heart disease						
No	80	94.10%	3575	98.00%	5.96	**.015**
Yes	5	5.90%	74	2.00%		

Data were presented as number (%).

Level of statistical significance, *P* < .05. The bold values indicates statistically significant.

* ,

† The same symbols indicate the non-significant difference between groups based on Bonferroni multiple comparison test.

### 7.2. Univariate analysis of general demographic characteristics

The proportion of stroke patients aged 65 to 79 years was significantly higher than that of participants aged 40 to 64 years. The proportion of stroke was also significantly higher in males than in females. Compared with participants with primary school education or below, those with high school or technical secondary school education had a significantly lower proportion of stroke (Table 1). The prevalence of comorbidities was significantly higher in stroke patients than in non-stroke participants, with statistical significance (all *P* < .05): hypertension (69.40% vs 36.90%), diabetes mellitus (30.60% vs 14.60%), dyslipidemia (68.20% vs 51.00%), coronary heart disease (5.9% vs 2.00%), atrial fibrillation (3.5% vs 2.3%), and carotid artery stenosis (21.20% vs 10.30%).

### 7.3. Univariate analysis of lifestyle factors between stroke patients and non-stroke participants

Significant differences were observed between stroke patients and non-stroke participants in terms of fruit and vegetable intake, salt taste preference, eating habits, physical activity level, BMI status, and smoking status (Table 2). Dietary patterns: The incidence of stroke was significantly higher in participants with a vegan diet than in those with a balanced meat-vegetable diet. Smoking status: Compared with current smokers and former smokers, nonsmokers accounted for a significantly lower proportion in the stroke group than in the non-stroke group (78.80% vs 91.10%, *P* = .001). Physical activity & BMI: The proportions of participants with physical inactivity and overweight were significantly higher in the stroke group than in the non-stroke group (physical inactivity: 30.60% vs 19.20%, *P* = .009; overweight: 81.20% vs 66.50%, *P* = .004). Fruit & vegetable intake: The proportion of participants with fruit/vegetable intake meeting the ≥100 g/day threshold for <2 days/week was significantly higher in stroke patients than in non-stroke participants (vegetables: 45.90% vs 28.70%, *P* = .014; fruits: 45.90% vs 28.70%, *P* = .003).

**Table 2:** Univariate analysis of lifestyle factor differences between stroke patients and non-stroke participants.

	Stroke		No-Stroke		χ^2^	*P*
Variable	n	%	n	%		
Salt taste preference						
Light	19*,†	22.40%	701*,†	19.20%	5.17	.75
Moderate	54†	63.50%	2655†	72.80%		
Salty preference	12*	14.10%	293*	8.00%		
Dietary patterns						
Vegan diet	26*	30.60%	784*	21.50%	12.824	**.002**
Balance of meat and vegetables	51†	60.00%	2731†	74.80%		
Meat diet	8*	9.40%	134*	3.70%		
Vegetable intake						
<2 d	44*	51.80%	1347*	36.90%	8.54	**.014**
3–4 d/wk	40†	47.10%	2280†	62.50%		
>5 d	1*,†	1.20%	22*,†	0.60%		
Fruit intake						
<2 d	39*	45.90%	1046*	28.70%	11.96	**.003**
3–4 d/wk	45†	52.90%	2538†	69.60%		
>5 d	1*,†	1.20%	65*,†	1.80%		
Physical exercise						
Lack of physical exercise	59	69.40%	2948	80.80%	6.86	**.009**
Regular physical exercise	26	30.60%	701	19.20%		
Drink						
Never drinking	73	85.90%	3197	87.60%	0.898	.638
Light drinking	9	10.60%	377	10.30%		
Heavy drinking	3	3.50%	75	2.10%		
Smoking						
Never smoker	67*	78.80%	3326*	91.10%	20.11	**.001**
Former smoker	4†	4.70%	35†	1.00%		
Current smoker	14†	16.50%	288†	7.90%		
Overweight						
No	16	18.80%	1224	33.50%	8.11	**.004**
Yes	69	81.20%	2425	66.50%		

Data were presented as number (%).

Level of statistical significance, *P* < .05. The bold values indicates statistically significant.

* ,

† The same symbols indicate the non-significant difference between groups based on Bonferroni multiple comparison test.

### 7.4. Multivariate analysis of the association between lifestyle factors and stroke risk

Table 3 presents the results of the regression analysis examining the association between lifestyle factors and stroke risk. After adjusting for confounding factors – including sex, age, educational level, annual household income, TC, TG, HDL-C, LDL-C, glycated hemoglobin, and homocysteine – adequate fruit and vegetable intake and a balanced meat-vegetable diet were found to have a protective effect against stroke. Dietary patterns: Compared with participants on a vegan diet, those with a balanced meat-vegetable diet had a 41% lower stroke risk [adjusted odds ratio (AOR) = 0.59, 95% CI: 0.36–0.97]. Fruit & vegetable intake: Compared with participants who met the ≥100 g/day intake threshold for <2 days/week, those who met this threshold for >5 days/week had a 57% lower risk of stroke for fruits (AOR = 0.43, 95% CI: 0.26–0.70) and a 55% lower risk for vegetables (AOR = 0.45, 95% CI: 0.27–0.76). Smoking, overweight, and physical inactivity were identified as independent risk factors for stroke: Compared with nonsmokers, smokers had a 1.18-fold higher stroke risk (AOR = 2.18, 95% CI: 1.04–4.57, *P* = .039). Compared with participants of normal weight, overweight participants had a 1.22-fold higher stroke risk (AOR = 2.22, 95% CI: 1.23–4.02, *P* = .019). Compared with participants who regularly engaged in physical activity, those with physical inactivity had a 75% higher stroke risk (AOR = 1.75, 95% CI: 1.05–2.93, *P* = .033).

**Table 3:** Multivariate logistic regression model analysis of the impact of lifestyle factors on stroke risk.

			Model 1	Model 2	Model 3
Variable	N	%	OR (95% Cl)	OR (95% Cl)	OR (95% Cl)
Taste preference					
Light	19	22.40%	1.00	1.00	1.00
Moderate	54	63.50%	0.75 (1.27–0.51)	0.83 (0.49–1.43)	0.82 (0.48–1.41)
Salty preference	12	14.10%	1.51 (0.72–3.15)	1.46 (0.69–3.08)	1.45 (0.69–3.07)
Eating habit					
Vegan diet	26	30.60%	1.00	1.00	1.00
Balance of meat and vegetables	51	60.00%	0.56 (0.35–0.91)	0.551 (0.24–1.27)	0.59 (0.36–0.97)
Meat diet	8	9.40%	1.8 (0.80–4.06)	0.33 (0.15–0.73)	1.80 (0.78–4.15)
Vegetable					
<2 d	1	1.20%	1.00	1.00	1.00
3–4 d/wk	39	47.10%	1.39 (0.18–10.56)	1.45 (0.18–11.42)	1.50 (0.19–11.80)
>5 d	45	51.80%	0.54 (0.35–0.83)	0.48 (0.29–0.79)	0.45 (0.27–0.76)
Fruit					
<2 d	1	1.20%	1.00	1.00	1.00
3–4 d/wk	45	52.90%	0.41 (0.06–3.95)	0.37 (0.05–2.76)	0.37 (0.05–2.75)
>5 d	39	45.90%	0.48 (0.3109.74)	0.44 (0.27–0.71)	0.43 (0.26–0.70)
Lack of physical exercise	26	30.60%	1.85 (1.16–2.96)	1.77 (1.09–2.87)	1.75 (1.05–2.93)
Drink					
Never drinking	73	85.90%	1.00	1.00	1.00
Light drinking	9	10.60%	1.05 (0.52–2.11)	0.59 (0.17–2.05)	0.73 (0.34–1.57)
Heavy drinking	3	3.50%	1.75 (0.54–5.68)	0.47 (0.12–1.82)	1.68 (0.48–5.84)
Smoking					
Never smoker	67	78.80%	1.00	1.00	1.00
Former smoker	4	4.70%	5.67 (1.96–16.41)	3.34 (1.09–10.27)	2.862 (0.80–10.31)
Current smoker	14	16.50%	2.41 (1.34–4.35)	2.03 (1.03–4.00)	2.18 (1.041–4.57)
Overweight	69	85.90%	2.18 (1.25–3.76)	2.13 (1.22–3.70)	2.22 (1.23–4.02)

Model 1: Did not adjust for any confounding factors; Model 2: Adjusted for gender, age, educational level, and annual income; Model 3: Based on Model 2, further adjusted for TC, TG, HDL, LDL, glycated hemoglobin, and homocysteine.

Level of statistical significance, *P* < .05.

CI = confidence interval, OR = odds ratio.

Different regression models were constructed based on variations in confounding factors. After adjusting for the aforementioned confounding factors, compared with participants with 0 to 1 unhealthy lifestyle factors, the stroke risk was 1.75-fold, 2.70-fold, and 22.67-fold higher in those with 2 to 3, 4 to 5, and ≥6 unhealthy lifestyle factors, respectively. The corresponding adjusted odds ratios (AORs) and 95% CIs were as follows: 2.745 (1.536–4.903), 3.704 (1.633–8.403), and 23.665 (6.434–87.051). A trend test showed statistical significance (*P* = .001), indicating a positive association between the number of unhealthy lifestyle factors and stroke risk (Table 4).

**Table 4:** Multifactorial analysis of the influence of the number of unhealthy lifestyles on stroke risk.

			Model 1	Model 2	Model 3
Unhealthy lifestyles	N of participants	N of stroke	OR (95% Cl)	OR (95% Cl)	OR (95% Cl)
0–1	1653	16	1.00	1.00	1.00
2–3	1785	54	3.19 (1.82–5.60)	2.82 (1.58–5.03)	2.75 (1.536–4.903)
4–5	274	11	4.28 (1.96–9.32)	3.86 (1.71–8.71)	3.70 (1.633–8.403)
>6	22	4	22.74 (6.92–74.74)	22.75. (6.25–82.86)	23.67 (6.434–87.051)
*P* for trend			.001	.001	.001

Model 1: Did not adjust for any confounding factors; Model 2: Adjusted for gender, age, educational level, and annual income; Model 3: Based on Model 2, further adjusted for TC, TG, HDL, LDL, glycated hemoglobin, and homocysteine.

The unhealthy lifestyle included 8 types: smoking, heavy drinking, lack of physical exercise, overweight, Low intake of fruits and vegetables, vegetarian or salty eating habits.

Level of statistical significance, *P* < .05.

CI = confidence interval, OR = odds ratio, *P* for trend = trend tests.

## 8. Sensitivity analyses

In sensitivity analyses, given that hypertension was identified as a risk factor for stroke, we conducted univariate and interaction analyses stratified by hypertension status (i.e., with and without hypertension) across different lifestyle subgroups. The results remained materially unchanged (see Files S1 and S2, Supplemental Digital Content, https://links.lww.com/MD/Q584).

## 9. Discussion

Lifestyle plays a far more critical role than many clinicians recognize. Dietary shifts in China driven by rising incomes are likely a key contributor to the significant increase in stroke risk over the past few decades – a trend that has accelerated in recent years. Specifically, consumption of meat and eggs has risen, while intake of fruits, vegetables, and whole grains has declined. According to the Global Burden of Disease Study 2019, China recorded 3.9 million new stroke cases, with stroke incidence increasing by 86.0% between 1990 and 2019.^[4]^ Fortunately, lifestyle interventions and early pharmacological treatment are effective in reducing stroke morbidity and mortality.^[27]^ In the U.S. Health Professionals Study and Nurses’ Health Study, poor lifestyle choices accounted for more than half of stroke cases.^[28]^ Participants who adhered to all 5 healthy lifestyle factors – nonsmoking, moderate alcohol consumption, a BMI < 25 kg/m^2^, 30 minutes of daily physical activity, and a healthy diet score in the top 40% – had an 80% lower stroke risk compared to those who did not. A study of Swedish women found that adherence to all 5 factors reduced stroke incidence by 60%,^[29]^ while among Swedish men with hypertension and dyslipidemia, adherence to these 5 beneficial behaviors reduced coronary events by more than 80%.^[29]^ These findings align with our results, which indicate a positive association between the number of unhealthy lifestyle factors and stroke risk.

In the present study, we observed no significant association between alcohol consumption (either light or heavy) and increased stroke risk compared with nondrinkers. This finding is consistent with the results of Suliga et al,^[30]^ who also reported no association between alcohol intake and stroke occurrence in their adjusted model. A meta-analysis by Larsson et al^[31]^ suggested that the effect of alcohol consumption on stroke incidence is dose-dependent: light alcohol intake may confer benefits, whereas heavy intake significantly increases stroke risk. This “double-edged sword” effect has been supported by subsequent studies: moderate alcohol consumption appears to reduce cardiovascular risk compared with abstinence, while heavy consumption elevates risk – particularly for stroke.^[32,33]^ Potential mechanisms include: heavy alcohol intake may increase blood pressure via sympathetic nervous system activation, and this adverse effect on blood pressure may directly raise the risk of hemorrhagic stroke^[34]^; conversely, moderate alcohol intake is associated with higher HDL-C levels, improved insulin sensitivity, and lower fibrinogen and inflammatory marker levels,^[35]^ which may partially reduce stroke risk. Thus, the relationship between alcohol consumption and stroke risk remains incompletely elucidated. Previous studies have indicated that the impact of alcohol intake on stroke risk is modified by factors such as sex, age, and chronic conditions, leading to inconsistent findings across different populations.^[36,37]^

In the present study, smoking, physical inactivity, and overweight were identified as independent risk factors for stroke: overweight was associated with an approximately 122% increased stroke risk in adults, physical inactivity with a 75%, ^[38]^ which induce structural changes in both arterioles and large arteries. These changes may lead to increased large-artery stiffness, as well as arteriolar white matter lesions and lacunar lesions – manifested on magnetic resonance imaging as detectable white matter degeneration, lacunar infarction, and cerebral microbleeds.^[39]^ Cigarette smoke contains numerous toxic substances, including nicotine and carbon monoxide, which can damage vascular endothelium and promote atherosclerotic plaque formation. These plaques narrow arterial lumens, reduce blood flow, and thereby increase the risk of ischemic stroke.^[40–42]^ Additionally, smoking elevates blood pressure and heart rate, further increasing stroke risk.^[43,44]^ Regular physical activity is crucial for maintaining cardiovascular health. Exercise improves blood circulation, lowers blood pressure, and helps maintain a healthy weight, all of which contribute to reducing stroke risk.^[45,46]^

While most studies support associations between smoking, obesity, and physical inactivity and increased stroke risk, some have reported conflicting findings. A study by O’Donnell et al^[47]^ found that the association between smoking and stroke risk may be weaker in certain populations – particularly in low- and middle-income countries – where other risk factors (e.g., poor diet, infectious diseases) may outweigh the effects of smoking.

Some studies suggest that the relationship between obesity and stroke risk is more complex than previously hypothesized. For instance, Berrington de González et al^[48]^ demonstrated that although obesity is generally a stroke risk factor, specific subgroups (e.g., older adults) may have a lower relative risk of stroke compared to obese younger individuals. Methodological variations across studies may contribute to inconsistent results. For example, self-reported physical activity measures are less accurate than objective assessments.^[49]^ Additionally, confounding factors – including diet, alcohol consumption, and socioeconomic status – may distort the associations between smoking, obesity, physical inactivity, and stroke risk. For example, failure to adjust for dietary factors may lead to overestimation of obesity’s impact on stroke risk.^[38]^ In conclusion, despite some inconsistencies in the literature, overwhelming evidence confirms that smoking, obesity, and physical inactivity are significant stroke risk factors. Further research is needed to clarify the nuances of these relationships, especially across diverse populations and contexts.

The present study confirmed that adequate fruit and vegetable intake is a protective factor for stroke. This finding is consistent with several recent studies that have supported the role of fruit and vegetable consumption in stroke prevention. For instance, a meta-analysis by Aune et al,^[50]^ which included 1.2 million participants from 95 studies, reported a 32% reduction in stroke risk among individuals consuming more than 5 servings of fruits and vegetables per day. These results align with our findings, highlighting the importance of fruit and vegetable intake for stroke prevention. While some studies have reported null associations, methodological variations and population-specific characteristics likely contribute to these discrepancies. However, such conflicting results underscore the complexity of dietary interventions and the need for standardized research methodologies. Future studies should focus on defining optimal intake levels of fruits and vegetables, improving dietary adherence, and exploring the synergistic effects of fruit and vegetable consumption with other lifestyle factors. Public health initiatives should continue to promote fruit and vegetable intake as part of a comprehensive stroke prevention strategy.

The relationship between dietary patterns and stroke risk has been extensively studied. Our findings indicate that individuals with moderate meat intake have a lower stroke prevalence compared to those adhering to a strict vegan diet. Several studies have suggested that vegan diets may reduce stroke risk, with proposed mechanisms including high intake of fruits, vegetables, and whole grains – foods rich in antioxidants, fiber, and essential nutrients. These components improve cardiovascular health by lowering blood pressure, optimizing lipid profiles, and reducing inflammation.^[45,46]^ Additionally, vegan diets are typically low in saturated fats and cholesterol, which are established stroke risk factors.^[51–53]^ For example, Satija et al^[52]^ found that a plant-based diet was associated with a lower risk of ischemic stroke, primarily attributed to its beneficial effects on blood pressure and cholesterol levels. Similarly, Wang et al^[51]^ reported that high fruit and vegetable intake – a hallmark of vegan diets – was linked to reduced stroke risk, underscoring the potential protective effects of vegan diets for stroke prevention. Conversely, our study highlights the protective role of moderate meat consumption against stroke, a finding consistent with other research exploring meat’s place in a balanced diet. Moderate meat intake provides essential nutrients such as high-quality protein, iron, zinc, and vitamin B12, which are critical for maintaining overall health and preventing nutritional deficiencies.^[54–56]^ Tong et al^[56]^ demonstrated that moderate consumption of lean meats (particularly poultry and fish) was associated with lower stroke risk, as omega-3 fatty acids (from fish) and high-quality protein (from lean meats) contribute to cardiovascular health. A prospective cohort study conducted by University of Oxford researchers also found that vegetarians and vegans had a higher risk of hemorrhagic stroke and total stroke compared to meat eaters, potentially due to low TC levels or insufficient intake of certain vitamins.^[57,58]^

## 10. Limitation

As with any survey-based analysis, several factors limit the generalizability of our findings. Firstly, data were collected from community-dwelling adults who were capable of participating in the physical examination and survey. Consequently, the results may not be representative of institutionalized individuals or those with functional limitations that precluded survey participation. Secondly, the survey did not collect data on stroke-specific features (e.g., hemiparesis, neglect, cognitive impairment). As a result, the potential moderating effect of these characteristics on lifestyle-related stroke risk factors could not be evaluated. Thirdly, the study used a cross-sectional design with self-reported data. Objective measurements and longitudinal data collection are needed to investigate temporal trends in lifestyle risk factors among individuals with stroke. Fourth, heavy drinking was defined exclusively based on white wine intake. A questionnaire design oversight resulted in the lack of systematic collection and classification of data on other low-alcohol beverages, which further limits the generalizability of our findings to broader populations.

## 11. Conclusion

Smoking, overweight, and physical inactivity are independent risk factors for stroke, while adequate fruit and vegetable intake and a balanced meat-vegetable diet act as protective factors. The present study aims to provide preliminary insights into the cumulative impact of these lifestyle factors on stroke risk among the target community population. Our study aims to provide preliminary insights into the collective impact of these lifestyle factors on stroke risk among our target community population. Additionally, our use of real-world, community-based data enhances the relevance of our findings to residents of similar urban communities in northern China, though their generalizability to more diverse demographic or geographic groups is limited. Future research should continue to explore optimal dietary patterns and lifestyle behaviors for stroke risk reduction, while also accounting for individual nutritional needs and preferences.

## Author contributions

**Conceptualization:** Qiang Zhou.

**Data curation:** Jiantao Yu, Jie Li, Qiang Zhou.

**Formal analysis:** Qiang Zhou.

**Funding acquisition:** Qiang Zhou.

**Investigation:** Jiantao Yu, Huiyong Yu.

**Methodology:** Jiantao Yu, Jie Li.

**Project administration:** Qiang Zhou.

**Resources:** Jiantao Yu, Jie Li.

**Software:** Jiantao Yu, Huiyong Yu.

**Supervision:** Jiantao Yu, Huiyong Yu.

**Validation:** Huiyong Yu.

**Visualization:** Jiantao Yu, Jie Li.

**Writing** – **original draft:** Qiang Zhou.

## Supplementary Material


