# Supplementary material for: Influence of lifestyle on stroke risk among adults over 40 years in northern China: A retrospective case-control study
Source: Medicine (Baltimore). 2025 Nov 7;104(45):e45707. doi: 10.1097/MD.0000000000045707 (PMC12599715; doi:10.1097/MD.0000000000045707)
Supplement: Supplementary file 1 [file medi-104-e45707-s001.docx]

**Table S1:** Multivariate regression analysis of the interaction between lifestyle factors and hypertension on stroke risk.

|  | Hypertension | Non-hypertension |  | Hypertension vs Non-hypertension |
| --- | --- | --- | --- | --- |
| Variables | N | N | P | OR(95%CL) |
| Taste preference |  |  |  |  |
| Light | 291 | 429 |  | 1.00 |
| Moderate | 980 | 1729 | 0.019 | 1.805（1.10-2.96） |
| Salty preference | 136 | 169 | <0.001 | 4.361（2.10-9.05） |
| Eating habit |  |  |  |  |
| Vegan diet | 351 | 459 |  | 1.00 |
| Balance of meat and vegetables | 972 | 1810 | 0.111 | 1.48（0.91-2.40） |
| Meat diet | 84 | 58 | <0.001 | 4.874（2.01-11.83） |
| Vegetable |  |  |  |  |
| <2day | 376 | 1016 | <0.001 | 4.295（2.44-7.58） |
| 3-4day/week | 1022 | 1298 | 0.059 | 1.78（0.98-3.24） |
| >5day | 9 | 13 |  | 1.00 |
| Fruit |  |  |  |  |
| <2day | 324 | 761 | <0.001 | 4.055（2.24-7.34） |
| 3-4day/week | 1062 | 1521 | 0.009 | 2.159（1.21-3.84） |
| >5day | 21 | 45 |  | 1.00 |
| Physical exercise |  |  |  |  |
| Lack of physical exercise | 1030 | 1977 |  | 1.00 |
| Regular physical exercise | 377 | 350 | <0.001 | 2.632（1.55-4.48） |
| Drink |  |  |  |  |
| Never drinking | 1123 | 2147 |  | 1.00 |
| Light drinking | 230 | 156 | 0.65 | 1.204（0.54-0.69） |
| Heavy drinking | 54 | 24 | 0.09 | 3.088（0.84-11.38） |
| Smoking |  |  |  |  |
| Never smoker | 1184 | 2209 |  | 1.00 |
| Former smoker | 27 | 12 | 0.071 | 3.293（0.91-11.99） |
| Current smoker | 196 | 106 | 0.117 | 1.842（0.86-3.95） |
| Overweight |  |  |  |  |
| NO | 381 | 859 |  | 1.00 |
| Yes | 1026 | 1468 | <0.001 | 2.966（1.85-4.76） |

Adjusted for gender, age, educational level, annual income, glycated hemoglobin, homocysteine and TC、TG、HDL、LDL.

Level of statistical signiffcance, P < 0.05.

CI, conﬁdence interval; OR, odds ratio;

**Table S2:** Univariate analysis of lifestyle factors in hypertensive and non-hypertensive participants

|  |  |  | Hypertension | Non-hypertension | chi-square | P |
| --- | --- | --- | --- | --- | --- | --- |
| Variables | n | % | N | % |  |  |
| Taste preference |  |  |  |  | 11.11 | 0.004 |
| Light | 291a, b | 20.70% | 429a，b | 18.40% |  |  |
| Moderate | 980b | 69.70% | 1729b | 74.30% |  |  |
| Salty preference | 136a | 9.70% | 169a | 7.30% |  |  |
| Eating habit |  |  |  |  | 47.81 | 0.001 |
| Vegan diet | 351a | 24.90% | 459a | 19.70% |  |  |
| Balance of meat and vegetables | 972b | 69.10% | 1810b | 77.80% |  |  |
| Meat diet | 84c | 6.00% | 58c | 2.50% |  |  |
| Vegetable |  |  |  |  | 107.66 | 0.001 |
| <2day | 376a | 26.70% | 1016a | 43.70% |  |  |
| 3-4day/week | 1022b | 72.60% | 1298b | 55.80% |  |  |
| >5day | 9a, b | 0.60% | 13a, b | 0.60% |  |  |
| Fruit |  |  |  |  | 42.19 | 0.001 |
| <2day | 324a | 23.00% | 761a | 32.70% |  |  |
| 3-4day/week | 1062b | 75.50% | 1521b | 65.40% |  |  |
| >5day | 21a, b | 1.50% | 45a, b | 1.90% |  |  |
| Physical exercise |  |  |  |  | 77.25 | 0.001 |
| Lack of physical exercise | 1030a | 73.20% | 1977a | 85.00% |  |  |
| Regular physical exercise | 377b | 26.80% | 350b | 15.00% |  |  |
| Drink |  |  |  |  |  |  |
| Never drinking | 1123a | 79.80% | 2147a | 92.30% | 127.45 | 0.001 |
| Light drinking | 230b | 16.30% | 156b | 6.70% |  |  |
| Heavy drinking | 54b | 3.80% | 24b | 1.00% |  |  |
| Smoking |  |  |  |  | 123.03 | 0.001 |
| Never smoker | 1184a | 84.20% | 2209a | 94.90% |  |  |
| Former smoker | 27b | 1.90% | 12b | 0.50% |  |  |
| Current smoker | 196b | 13.90% | 106b | 4.60% |  |  |
| Overweight |  |  |  |  | 38.24 | 0.001 |
| NO | 381a | 27.10% | 859a | 36.90% |  |  |
| Yes | 1026b | 72.90% | 1468b | 63.10% |  |  |

Data were presented as number (%) .

^a, b^ the same letters indicate the non-signiﬁcant diﬀerence between groups based on Bonferroni multiple comparison test.

Level of statistical signiffcance, P < 0.05.
